# Assessing ITN textile preferences: A comparative study of polyethylene and polyester nets across different settings in Burkina Faso

**DOI:** 10.1371/journal.pone.0325580

**Published:** 2025-08-22

**Authors:** Hervé Hien, Aristide S. Hien, Fidèle Y. Bacyè, Hermann Badolo, Alfred Tiono, Cheick O. Diallo, Serge M.A. Somda, Hermann Bazié, Matilibou Guira, Nicolas Meda

**Affiliations:** 1 Institut de Recherches en Sciences de la Santé, Centre national de la recherche scientifique et technologique, Ouagadougou, Burkina Faso; 2 Institut National de Santé Publique, Ouagadougou, Burkina Faso; 3 Centre Universitaire de Tenkodogo, Tenkodogo, Burkina Faso; 4 Centre National de Formation et de Recherche sur le Paludisme, Ouagadougou, Burkina Faso; 5 Université Nazi Boni, Bobo-Dioulasso, Burkina Faso; 6 Ministère de la santé et de l’hygiène publique, Programme d’Appui au Développement Sanitaire, Ouagadougou, Burkina Faso; 7 Université Joseph Ki-Zerbo, Unité de Formation et de Recherches en Sciences de la Santé, Ouagadougou, Burkina Faso; University of Uyo, NIGERIA

## Abstract

Insecticide-treated nets (ITNs) are crucial for preventing malaria and other vector-borne diseases, effectively reducing transmission and mortality rates. However, their uptake remains low, influenced by textile preferences, with polyester nets being favored for comfort, despite their shorter lifespan compared to other materials. A study in Burkina Faso aimed to understand community preferences for ITN fabrics across different environmental contexts to improve malaria control strategies. Using a mixed-methods approach, the study surveyed and interviewed urban and rural communities in three climatic zones of Burkina Faso to identify socio-economic, environmental, and cultural factors shaping ITN material preferences. The study found that 95.2% (95% CI: 94.6–95.7) of participants had a preference for a specific ITN fabric, with 93% (95% CI: 92.3-93.6) favoring polyester over polyethylene, citing superior comfort, breathability, and protection. Regional differences emerged, with rural areas and the Humid savannah-Sahelian zone showing a stronger preference for polyester nets, especially in larger sizes. The study recommended that malaria control programs prioritize polyester-based nets, offer larger sizes for rural populations, use smaller mesh sizes, and adopt rectangular designs to improve coverage and comfort. Urban populations emphasized comfort and efficacy, while rural areas focused more on durability and size. Factors like ease of maintenance, comfort, and local climate were significant in shaping preferences. The study concluded that tailoring ITN distribution strategies to regional needs such as adjusting net size and mesh could improve malaria control efforts. These insights can guide the optimization of malaria prevention programs, enhancing their effectiveness and long-term sustainability in diverse settings.

## Introduction

Insecticide-treated nets (ITNs) remain a cornerstone of global malaria control efforts, offering dual protection through both physical barrier effects and insecticidal activity against *Anopheles* vectors [1,2]. The World Health Organization (WHO) recommends ITNs as a first-line intervention, and their widespread deployment has contributed substantially to the decline in malaria morbidity and mortality over the past two decades [1,3]. Meta-analyses and large-scale trials have demonstrated that ITNs can reduce malaria incidence by up to 50% and significantly lower child mortality in high-transmission settings [2,4,5]. Moreover, ITNs confer ancillary benefits in reducing the transmission of other mosquito-borne diseases such as lymphatic filariasis, particularly in regions with vector species overlap [6–8]. Despite these proven public health gains, the long-term effectiveness of ITN-based strategies faces several implementation challenges. Chief among them is the increasing prevalence of pyrethroid resistance in mosquito populations, which threatens the entomological efficacy of conventional ITNs [9–12]. In response, next-generation ITNs incorporating synergists such as piperonyl butoxide (PBO) or combining multiple active ingredients (e.g., chlorfenapyr, pyriproxyfen) have been introduced to mitigate resistance-related declines in vector control performance [13–17]. However, while the entomological efficacy of these innovations is well-documented, their operational success ultimately hinges on consistent and widespread community use.

A growing body of evidence suggests that the physical properties of ITNs especially textile type play a critical role in determining user acceptability, adherence, and nightly usage [18–21]. In hot and humid environments typical of sub-Saharan Africa, perceived comfort, breathability, and ease of maintenance are frequently cited as key factors influencing ITN use [22]. Polyethylene ITNs, although more durable, are often perceived as rough, stiff, and less breathable. In contrast, polyester ITNs are softer, more pliable, and better ventilated but tend to degrade faster [23]. When ITNs are perceived as uncomfortable or deteriorate quickly, users may stop using them altogether undermining the community-wide coverage required for sustained malaria prevention [18,24].

In Burkina Faso, five national ITN mass distribution campaigns have been conducted since 2010, leading to high coverage rates [25]. However, usage remains inconsistent: in 2021, only 61% of the population reported sleeping under an ITN the previous night [26,27]. Usage patterns also vary significantly across ecological zones, with some regions reporting less than 50% coverage [26]. Retrospective assessments, including reports from the Alliance for Malaria Prevention, have highlighted temporal fluctuations in ITN fabric preferences, with polyethylene ITNs being more commonly used in some areas as recently as 2018, though by 2021, no clear textile preference was evident at the national level [28]. However, these national surveys lack granularity, ecological stratification, and behavioral depth. In addition, Hien et al. [27] described a protocol designed to generate high-quality, prospective, and contextually relevant scientific data using a rigorous methodological approach. The manuscript also emphasized the importance of collecting stratified contextual data across different regions of the country to inform evidence-based decision-making. Moreover, this paper, have discussed on gaps related to using retrospective data collection, without adequately exploring the social, environmental, and operational factors that shape textile preferences and their implications for ITN usage. As new-generation ITNs are increasingly deployed, it is essential to ensure not only their entomological efficacy but also their physical acceptability and consistent use within target communities. Without this alignment, technological advancements may fail to achieve their intended public health impact. To address this critical gap, the present study conducted a large-scale, prospective assessment of ITN textile preferences across Burkina Faso. By incorporating both quantitative and qualitative data from urban and rural households within three distinct ecological zones Humid savannah, Dry savannah-Sahelian, and Sahelian, the study aimed to capture the complex socio-environmental factors influencing ITN acceptability and use. The specific objectives were to: i) assess community-level preferences between polyethylene and polyester ITNs; ii) examine variations in textile preferences across ecological zones and residential contexts (urban vs. rural); and iii) provide evidence-based recommendations to guide the National Malaria Control Program (NMCP) and ITN donors in aligning procurement and distribution strategies with user needs and behavioral determinants.

## Methods

### Study design

This study employed a cross-sectional, quasi-experimental design integrating both quantitative and qualitative approaches to assess community preferences for two ITN textile types—polyethylene and polyester—across three ecological zones in Burkina Faso: Sahelian, Dry Savannah (hereafter referred to as Sudano-Sahelian), and Humid Savannah. The study was conducted between January 31 and June 30, 2023, with household recruitment occurring from February 10 to 24, 2023. The primary aim was to examine user preferences and determinants influencing the use of ITNs based on textile type, considering both individual- and community-level factors in rural and urban settings.

A mixed-methods framework was used to provide a comprehensive understanding of ITN textile preferences [27]. Quantitative data measured the scale and determinants of textile preferences across geographic and demographic strata, while qualitative methods explored the underlying perceptions, experiences, and contextual factors influencing net usage behavior. Participants were shown labeled photographs and physical samples of polyethylene and polyester ITNs, which were matched in shape and color to reduce visual bias. Brand identifiers were removed to minimize manufacturer influence.

### Study area

**Fig 1:**
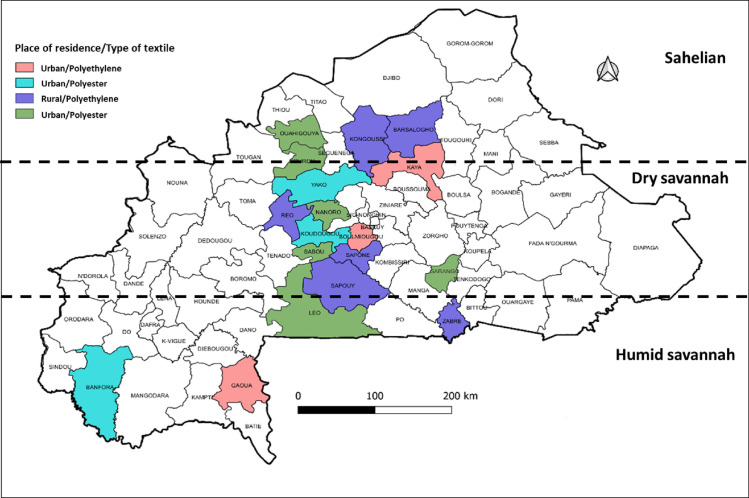
Geographic distribution of study sites across climate areas in Burkina Faso.

The study was conducted across 18 health districts, stratified by ecological zone (6 per zone) and place of residence (2 urban and 4 rural districts per zone). The selected zones are characterized as follows (Fig 1):

- Humid Savannah: High rainfall and dense vegetation, with a prolonged rainy season.
- Dry Savannah (Sudan-Sahelian): Transitional zone with moderate rainfall and marked seasonal variation.
- Sahelian: Semi-arid region with high temperatures and low, irregular rainfall.

Within each ecological zone, study communities were selected to represent the diversity of socioeconomic and environmental conditions affecting ITN usage.

### Study population and sampling

The study population comprised adult residents (≥18 years) in randomly selected households from both urban and rural communities across the three ecological zones. A multistage stratified random sampling approach was applied [27]:

1. Stratification by ecological zone and residence type (urban/rural);
2. Random selection of districts and enumeration areas;
3. Systematic sampling of households within each area.

Household inclusion criteria were: permanent residency in the selected district and willingness to provide written informed consent. Community Health Workers (CHWs) introduced the study and trained field staff obtained informed consent from all participants.

A total of 3,780 households were surveyed, with approximately 1,260 households per ecological zone. Based on the national average household size of 5.2 individuals and the ITN allocation ratio of one net per two people (as per GCPH 2019), the study encompassed approximately 9,450 ITNs.

### Quantitative data collection

A structured questionnaire was administered to heads of households and key members. The tool captured [27]:

- Demographic variables: age, sex, education level, household size, and socioeconomic status.
- Textile preference indicators: participants preferred ITN material (polyethylene or polyester). Participants were asked to rate their preferences for different ITN characteristics (e.g., texture, breathability, ease of maintenance) using a 3-point Likert scale: 1 = Not important, 2 = Moderately important, and 3 = Very important.
- Respondents identified key reasons for their preferences (e.g., comfort, breathability, ease of maintenance, durability, perceived efficacy).

### Qualitative data collection

#### Focus Group Discussions (FGDs).

Twelve focus group discussions (FGDs) four per ecological zone—were conducted to explore shared attitudes toward ITN textiles. Each FGD comprised 8–12 participants, selected to ensure a balanced representation of gender, age groups, and household types. Discussions were held in both urban and rural districts and guided by a semi-structured framework covering four main themes: (1) textile comfort and breathability, (2) perceived durability and ease of use, (3) cultural acceptability, and (4) perceptions of protective efficacy (S1). All discussions were conducted in local languages commonly spoken in the study areas primarily Dioula and Mooré depending on the participants’ linguistic background. Trained local facilitators who were fluent in these languages and familiar with the cultural context led the sessions. No external interpreters were needed, as the facilitators also served as translators when necessary and ensured accurate communication throughout the discussions. All session were audio-recorded with the informed consent of all participants.

#### In-depth Interviews (IDIs).

Twenty-four IDIs were conducted with CHWs and selected household representatives from each ecological zone. Interviewees were purposively selected based on their expressed preferences or unique perspectives during FGDs. Interviews explored:

- personal experiences with ITN materials;
- cultural and environmental influences on preference;
- suggested improvements to ITN design and distribution.

All interviews followed a semi-structured guide and were conducted in local languages with translation when necessary (S2).

### Ethics approval and consent to participate

The study was conducted in accordance with the ethical principles of health research and good clinical practice, as well as the guidelines set forth in the Declaration of Helsinki. The study protocol was reviewed and approved by the Institutional Ethics Committee (IEC) of the Institut National de Santé Publique (INSP), receiving ethical approval under the reference number 2023–001/MSHP/SG/INSP/CEI. Furthermore, field data collection was authorized by the Ministry of Health. Written informed consent was obtained from all participants prior to the commencement of any activities.

### Data analysis

#### Quantitative analysis.

Data were entered into a secure database and analyzed using R (version 4.2.2). Descriptive statistics (frequencies, means, proportions) summarized ITN preferences across zones and residence types. Bivariate and multivariate logistic regression models were used to identify predictors of preference for polyester versus polyethylene ITNs, controlling for demographic and contextual factors. A 5% significance level was used for all tests.

#### Qualitative analysis.

Audio recordings from FGDs and IDIs were transcribed verbatim and translated into French. An inductive coding framework was applied using NVivo software. Thematic analysis was conducted in five steps:

1. Initial coding of concepts and patterns;
2. Development of themes from codes;
3. Reviewing and refining themes for consistency;
4. Defining and naming themes relevant to ITN textile choice;
5. Interpretation of themes in relation to research objectives and quantitative findings.

## Results

### Sociodemographic profile of respondents

A total of 3,780 households were surveyed across the three climatic zones, Sahelian, Dry savannah, and Humid Savannah—with equal distribution (33.3%) per zone (Table 1). The majority of respondents (67.7%; n = 4164) resided in rural areas, compared to 32.3% (n = 1988) in urban settings. Statistically significant differences were observed in age distribution between zones, particularly within the 18–35 age group (χ² = 12.73, df = 2, p = 0.004), with the Humid Savannah zone having the highest proportion of younger respondents. Educational attainment varied across zones: the proportion of individuals with no formal education was highest in the Dry savannah zone (χ² = 52.9, df = 6, p < 0.001). Wealth quintile distributions also differed significantly across zones (χ² = 61.3, df = 8, p < 0.001), with the “very poor” category more frequent in the Humid Savannah-Sahelian region (Table 2).

**Table 1:** Distribution of surveyed households by climate zone and residence place.

	Total number	Proportions (%)
**Climate zone**		
Sahelian	1260	33,3
Dry savannah	1260	33,3
Humid savannah	1260	33,3
**Place of residence**		
Urban	1260	33,3
Rural	2520	66,7
**Overall**	**3780**	**100**

**Table 2:** Sociodemographic characteristics of participants across climate zones.

Variables	Climate areas (n, %)				
	Sahelian	Dry savannah	Humid savannah	Total	*P-value*
**Age group**					
18-35	1227 (59,2)	1205 (56,9)	1226 (62,5)	3658 (59,5)	0,004
35-45	521 (25,2)	588 (27,8)	480 (24,5)	1589 (25,8)	
45-60	323 (15,6)	324 (15,3)	256 (13,0)	903 (14,7)	
**Education status**					
None	1058 (51,3)	1151 (54,4)	1009 (51,5)	3218 (52,4)	<0,001
Primary/Post-primary	676 (32,8)	620 (29,3)	684 (34,9)	1980 (32,3)	
Secondary	259 (12,6)	209 (09,9)	198 (10,1)	666 (10,9)	
Higher	70 (03,4)	136 (06,4)	67 (03,4)	273 (04,4)	
**Gender**					
Male	883 (42,6)	930 (43,9)	804 (41,0)	2617 (42,5)	0,163
Female	1190 (57,4)	1187 (56,1)	1158 (59,0)	3535 (57,5)	
**Place of residence**					
Urban	656 (31,6)	650 (30,7)	682 (34,8)	1988 (32,3)	0,016
Rural	1417 (68,4)	1467 (69,3)	1280 (65,2)	4164 (67,7)	
**Wealth quintile**					
Very poor	602 (29,0)	1062 (50,2)	658 (33,5)	2322 (37,7)	<0,001
Poor	868 (41,9)	608 (28,7)	808 (41,2)	2284 (37,1)	
Average	465 (22,4)	275 (13,0)	382 (19,5)	1122 (18,2)	
Wealthy	124 (06,0)	132 (06,2)	102 (05,2)	358 (05,8)	
Very wealthy	14 (00,7)	40 (01,9)	12 (00,6)	66 (01,1)	
**Overall**	**2073 (33.7)**	**2117 (34.4)**	**1962 (31.9)**	**6152 (100,0)**	

### Overall ITN textile preference

**Fig 2:**
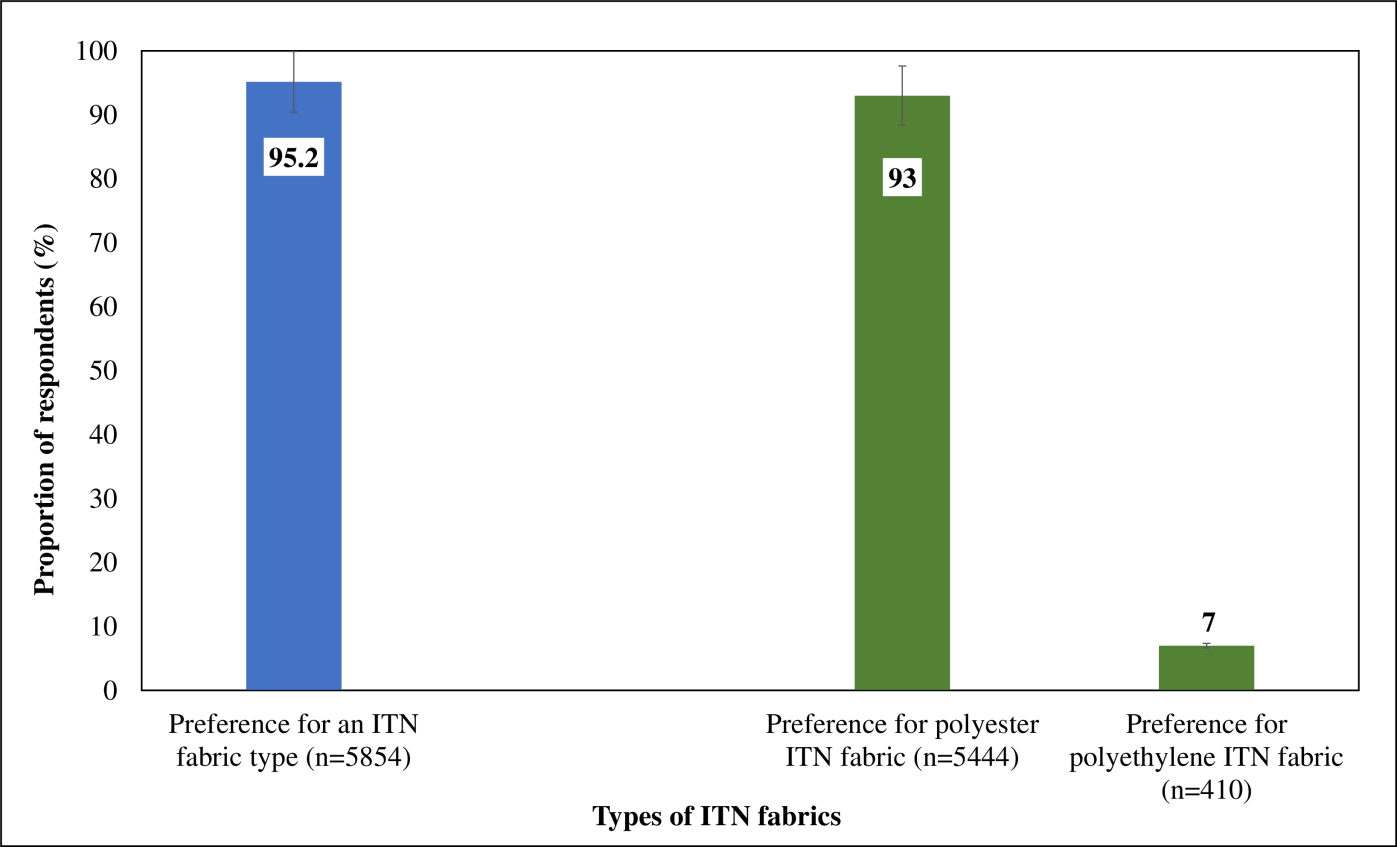
Global fabric preference among ITN users (polyester vs. polyethylene).

Among the 6,152 respondents who were shown matched samples of polyester and polyethylene ITNs, 95.2% (n = 5854; 95% CI: 94.6–95.7) expressed a clear preference for one fabric type. Polyester was overwhelmingly preferred by 93% (n = 5444; 95% CI: 92.3–93.6) of respondents, while only 7% (n = 410; 95% CI: 6.4–7.7) favored polyethylene (χ² = 4642.1, df = 1, p < 0.001) (Fig 2).

Qualitative findings from focus group discussions (FGDs) and in-depth interviews (IDIs) supported the quantitative results. Comfort and effectiveness against mosquito bites were the primary reasons for choosing polyester:

“*Polyester nets seem to keep the mosquitoes out more effectively. When I use them, I rarely get bitten*.” – FGD, Sahelian zone

“*Polyester is softer. The polyethylene ones feel too rough to sleep under*.” – IDI, Urban area

### Preference by climate zone and residence

Textile preference was consistent across all climatic zones, although modest variations were observed (χ² = 4.84, df = 2, p = 0.089). In contrast, rural residents showed significantly higher preference for polyester nets than urban residents (χ² = 253.7, df = 1, p < 0.001) (Table 3). Logistic regression analysis confirmed that rural residence (OR = 68.48, 95% CI: 67.64–71.82) and female gender (OR = 24.74, 95% CI: 21.42–31.76) were independently associated with polyester preference.

**Table 3:** Distribution of ITN textile preferences by climatic zone and residence.

Variables	Socio-demographics parameters	% (n = participants)	*P-value Intra group*	*P-value Inter group*
Preference for an ITN fabric type	Yes	95.2 (5854)	<0.001	NA
	No	4.8 (298)		
Preference for polyester fabric (soft fabric)	Humid savannah	31.8 (1731)	0.089	<0.001
	Dry savannah	33.3 (1814)		
	Sahelian	34.9 (1899)		
	Urban	32.3 (1757)	<0.001	
		Rural	67.7 (3687)	
Preference for polyethylene fabric (hard fabric)	Humid savannah	38.8 (159)	0.032	
	Dry savannah	37.3 (153)		
	Sahelian	23.9 (98)		
	Urban	33.2 (136)	<0.001	
		Rural	66.8 (274)	
Preference for larger sizes (width)	Humid savannah	30.8 (1803)	<0.001	NA
	Dry savannah	27.7 (1621)		
	Sahelian	41.5 (2429)		
	Urban	32.6 (1908)	<0.001	
		Rural	67.4 (3945)	
Preference for larger sizes (height)	Humid savannah	35.1 (2055)	<0.001	
	Dry savannah	30.6 (1791)		
	Sahelian	34.4 (2014)		
	Urban	29.1 (1703)	<0.001	
		Rural	70.9 (4150)	
Preference for smaller mesh sizes	Humid savannah	43.0 (2517)	<0.001	<0.001
	Dry savannah	34.7 (2031)		
	Sahelian	22.3 (1305		
	Urban	42.1 (2464)	<0.001	
		Rural	57.9 (3389)	
Preference for larger mesh sizes	Humid savannah	47.9 (2804)	0.9	
	Dry savannah	24.9 (1458)		
	Sahelian	27.2 (1592)		
	Urban	26.4 (1545)	0.019	
		Rural	73.6 (4308)	
Preferences for rectangular-shaped	Humid savannah	34.7 (2031)	<0.001	<0.001
	Dry savannah	56.1 (3284)		
	Sahelian	9.2 (538)		
	Urban	36.7 (2148)	<0.001	
		Rural	63.3 (3705)	
Preferences for conical-shaped	Humid savannah	21.4 (1253)	<0.001	
	Dry savannah	36.5 (2137)		
	Sahelian	42.1 (2464)		
	Urban	36.6 (2142)	<0.001	
		Rural	63.4 (3712)	

FGDs and IDIs revealed consistent themes across climate zones. Participants cited breathability and heat tolerance as critical, particularly in humid regions:

“*In the humid climate, polyester nets feel cooler. Polyethylene traps heat.”* – FGD, Humid Savannah-Sahelian

“*Polyester dries faster when washed. Polyethylene stays wet longer*.” – IDI, Rural area

### Preference for ITN design features

Preferences extended beyond fabric to net design. Participants in the Sahelian zone showed the strongest preference for larger-sized ITNs (χ² = 32.6, df = 2, p < 0.001), with rural households favoring wider and taller nets to accommodate larger family sizes. Mesh size preferences also varied by region: preference for smaller mesh was highest in the Humid savannah zone (43.0%) and lowest in the Sahelian region (22.3%) (χ² = 29.5, df = 2, p < 0.001). Rural residents showed stronger preference for smaller mesh compared to urban counterparts (χ² = 17.9, df = 1, p < 0.001).

### Determinants of polyester ITN preference

Multivariate logistic regression analysis (Table 4) identified several significant predictors of polyester preference. Female participants were 24.74 times more likely than males to prefer polyester (95% CI: 21.42–31.76, p < 0.001). Age was another strong determinant: respondents aged 35–45 (OR = 5.91, 95% CI: 4.24–7.37, p < 0.001) and 45–60 (OR = 14.23, 95% CI: 12.71–15.40, p < 0.001) were significantly more likely to favor polyester than those aged 18–35. Wealth status also influenced preference, with poorer quintiles significantly more likely to prefer polyester (p < 0.001). Climate zones influenced preference as well: residents of Dry savannah zone were more likely to prefer polyester than those in the Sahelian zone (p < 0.001).

**Table 4:** Logistic regression analysis of determinants of polyester ITN preference.

Socio-demographic parameters	Crude Odds Ratio [95% CI]	P-value
**Gender**		
Male	–	
Female	24,74 [21.42–31.76]	<0,001
**Age group**		
[18-35]	–	
[35-45]	5,91 [4.24–7.37]	<0,001
[45-60]	14,23 [12.71–15.4]	<0,001
**Wealth quintile**		
Very poor	–	
Poor	55,09 [52.24–59.83]	<0,001
Average	214,43 [211–218.12]	<0,001
Wealthy	348,97 [301.76–350.76]	<0,001
Very wealthy	137,69 [135.42–141.23]	0,008
**Climate areas**		
Sahelian	–	
Dry savannah	20,70 [0.42–0.76]	<0,001
Humid savannah	8,95 [7.72–9.34]	<0,001
**Place of residence**		
Urban	–	
Rural	68.48 [67.64–71.82]	<0,001

### Community recommendations for ITN distribution programs

Participants across all climate zones recommended polyester nets due to their comfort, breathability, and ease of maintenance. They also favored rectangular shapes, larger dimensions for shared beds, and fine mesh for greater protection:

“*Polyester is breathable and easier to maintain—it fits our climate*.” – IDI, Rural Sahelian

“*Rectangular nets are better for family beds and easier to hang*.” – FGD, Urban Humid Savannah

Based on community input, the following design and distribution priorities are recommended (Table 5):

**Table 5:** Summary of preferred ITN fabric and design characteristics by community consensus.

ITNs characteristics	Recommendation for National Vector-Borne Disease Programs	Recommendation for ITN Donors
Type of ITN Fabrics	**Polyester ITNs fabrics**	
	Promote polyester ITNs as they offer better protection against mosquitoes, helping to improve malaria prevention efforts.	Support the distribution of polyester-based ITNs to enhance protection against mosquitoes, especially in endemic areas.
	Ensure that ITNs are made of comfortable, breathable materials like polyester to encourage consistent use by communities.	Focus on polyester nets, which are perceived as more comfortable for regular use, to improve adherence to malaria control measures.
ITN Size	**High width and height ITNs**	
	Provide larger nets, especially for rural areas with larger family structures, to ensure that all family members are protected.	Offer high-width and high-height ITNs to accommodate families and ensure effective malaria prevention in larger spaces.
ITN Mesh Size	**Smaller mesh size**	
	Promote the use of ITNs with smaller mesh sizes to provide superior protection against malaria vectors.	Distribute ITNs with smaller mesh sizes to offer enhanced protection against mosquitoes, particularly in malaria-endemic areas.
ITN Form	**Rectangular form**	
	Encourage the distribution of rectangular ITNs, as they are easier to set up and provide better coverage for family beds.	Provide rectangular ITNs to ensure they fit most bed sizes, maximizing their utility and effectiveness in both urban and rural households.
	Ensure rectangular ITNs are made widely available in both urban and rural markets, leveraging familiarity with this design.	Support the supply of rectangular ITNs, which are widely recognized and trusted by communities for their practicality and ease of use.

- Polyester as the preferred material
- Rectangular shape for practicality
- Larger dimensions for rural households
- Fine mesh for increased protection

## Discussion

This study provides a comprehensive analysis of community preferences for insecticide-treated net (ITN) fabrics specifically polyester and polyethylene across ecological zones and urban–rural settings in Burkina Faso, offering valuable evidence to inform user-centered vector control strategies. Employing a mixed-methods approach, the study examined how physical, socio-demographic, and environmental factors influence perceptions and decisions surrounding ITN use [19,29]. A striking finding is the overwhelming preference for polyester nets, reported by 93% of respondents, a trend consistent across eco-climatic and residential settings, and particularly strong in rural areas. Qualitative data supported this preference, with participants highlighting polyester’s comfort, softness, breathability, ease of washing, and rapid drying—attributes well suited to hot and humid climates and frequently cited in the literature [19,30–32]. These results challenge prevailing assumptions that polyethylene nets are preferred in rural or low-income areas due to their durability [23,33]. On the contrary, rural residents demonstrated stronger preferences for polyester (OR = 68.48), suggesting that perceived comfort and usability may outweigh durability, especially in environments where nets are subject to frequent washing and wear. In addition, socio-demographic factors also influenced fabric preference. Women were significantly more likely than men to prefer polyester (OR = 24.74), likely reflecting their central role in household health and caregiving. Older adults, particularly those aged 45–60, also favored polyester, possibly due to heightened health awareness or comparative experience with both materials. These patterns underscore the importance of gender- and age-sensitive approaches in ITN policy and programming [18, 29,34]. Socio-economic status was another determinant, with respondents in the “poor” and “average” wealth quintiles more likely to prefer polyester than those in the “very poor” or “very wealthy” groups, indicating a nonlinear relationship between wealth and fabric preference [29, 35]. Climatic conditions also played a role; residents of the Humid savannah and Humid savannah–Sahelian zones expressed greater preference for polyester than those in the drier Sahelian region, likely due to polyester’s superior breathability and comfort in more humid environments [18,36].

Beyond fabric type, respondents expressed preferences for other ITN design features. Larger nets were favored in the Sahelian zone, likely reflecting larger household sizes. Smaller mesh size was particularly valued in rural areas, where protection against small-bodied vectors is a concern, supporting recommendations for finer mesh ITNs in high-transmission, insecticide-resistant areas [19]. Rectangular nets were preferred across all settings, with participants citing ease of installation, better coverage, and compatibility with family-sized sleeping arrangements as key advantages findings consistent with previous studies linking net shape to use [22]. Qualitative data reinforced these findings, with participants frequently describing polyethylene nets as rough, uncomfortable, or irritating, and praising polyester for its softness, skin compatibility, and cooler feel during hot nights. These narratives add contextual depth to the quantitative trends and emphasize that fabric preferences are not merely aesthetic, but shaped by behavioral, environmental, and socio-economic realities. Failure to consider these preferences in procurement and distribution may undermine adherence, even when nets are bioeffective.

Overall, the findings highlight the need to align ITN distribution strategies with community preferences. Current procurement often emphasizes cost-effectiveness and durability, but this study shows that prioritizing comfort and usability especially by providing polyester nets could significantly enhance nightly ITN use. Programs should consider offering diverse ITN options that incorporate user-preferred features, such as larger size, rectangular shape, and finer mesh, and tailor distribution based on ecological zone, family size, and residential context. The strong rural preference for polyester, which contradicts existing policy assumptions, should inform national ITN procurement guidelines moving forward.

Several limitations merit consideration. The cross-sectional design limits causal inference between textile preference and long-term ITN use behavior, highlighting the need for longitudinal studies. Additionally, social desirability bias may have influenced responses in qualitative sessions, and while the sample was stratified by ecological and residential context, further disaggregation (e.g., by ethnicity, sleeping practices, or market access) could yield deeper insights. Despite these limitations, the study provides robust, policy-relevant evidence that aligning ITN procurement with user preferences can enhance adherence and contribute to more effective malaria control.

## Conclusion

This study provides robust, ecologically stratified evidence that polyester ITNs are overwhelmingly preferred by users in Burkina Faso due to their comfort, breathability, and ease of maintenance. These preferences transcend urban–rural and climate divides, challenging conventional procurement priorities that emphasize durability. To improve ITN effectiveness, national programs and donors should prioritize the distribution of polyester-based, rectangular, larger-sized nets with finer mesh. Such user-centered approaches are essential to enhance nightly adherence, increase community coverage, and maximize the impact of ITNs in malaria-endemic regions.

## Supporting information

S1 Focus Group Discussion (FGD) Guide and Questionnaire: Community-Level Insights on ITN textile (materials). (DOCX)

S2 In-Depth Individual Interview (IDI) Guide and questionnaire: Personal Experiences and Suggestions for ITN Improvements. (DOCX)
