# Supplementary material for: Assessing ITN textile preferences: A comparative study of polyethylene and polyester nets across different settings in Burkina Faso
Source: PLoS One. 2025 Aug 22;20(8):e0325580. doi: 10.1371/journal.pone.0325580 (PMC12373252; doi:10.1371/journal.pone.0325580)
Supplement: S1 — (DOCX) [file pone.0325580.s001.docx]

### **Additional file 1.** **Focus Group Discussion (FGD) Guide and Questionnaire: Community-Level Insights on ITN textile (materials)**

**Objective:**
To explore community-level preferences, perceptions, and experiences regarding the use of polyethylene and polyester insecticide-treated nets (ITNs), particularly focusing on material preference, comfort, durability, maintenance, and perceptions of efficacy.

**Introduction (10-15 minutes)**

1. **Welcome and Introductions:**
   - Welcome participants and briefly explain the purpose of the FGD.
   - Explain the importance of participants’ views in improving public health interventions.
   - Clarify that all opinions are valuable and that there are no right or wrong answers.
   - Assure confidentiality and explain how the data will be used.
2. **Ground Rules:**
   - Encourage everyone to speak openly and respectfully.
   - Emphasize that everyone’s opinion matters.
   - Note that discussions will be guided but may flow naturally based on participants’ responses.
3. **Icebreaker Question :**
   - *“Can everyone introduce themselves and briefly tell us how long you’ve been using insecticide-treated nets (ITNs) in your home?”*

**Main Discussion (40-60 minutes)**

**Discussion Point i: Material Preference (15-20 minutes)**

**Objective:** Understand which material (polyethylene or polyester) participants prefer and why, focusing on perceived benefits and drawbacks.

1. *“What type of insecticide-treated net do you currently use in your home—polyethylene or polyester? Why did you choose that one ?”*
2. *“Can you describe what you like most about the material you use (polyethylene or polyester)?”*
3. *“What do you think are the biggest advantages of the net material (polyethylene or polyester)? Why?”*
4. *“Have you ever used both polyethylene and polyester nets? If yes, how do they compare in your experience?”*
5. *“Do you believe one material is better than the other in terms of keeping mosquitoes away? Why or why not ?”*

**Discussion Point ii:** Comfort and Usability (15-20 minutes)

**Objective:** Understand how climate factors influence participants’ preferences regarding the comfort, texture, breathability, and warmth of each textile type*.*

1. *“How comfortable are the nets when you sleep under them? Do you feel the material affects your sleep in any way?”*
2. *“How would you describe the texture of the polyethylene and polyester nets? Are they rough or soft to the touch?”*
3. *“What about breathability? Do you feel that one material allows for better air circulation than the other?”*
4. *“In your climate (e.g., hot and dry, humid), do you find one material more comfortable than the other to sleep under?”*
5. *“Do you feel the net material (polyethylene or polyester) keeps you warmer or cooler? How does this affect your choice of material?”*

**Discussion Point iii:** Durability and Maintenance (15-20 minutes)

**Objective:** Explore how participants perceive the durability, maintenance, and lifespan of both polyethylene and polyester ITNs.

1. *“How long do your ITNs typically last before you need to replace them? Do you think the material (polyethylene or polyester) affects their lifespan?”*
2. *“Have you experienced any issues with your ITNs tearing, wearing out, or losing their effectiveness? Which material do you think is more prone to damage?”*
3. *“How easy is it to clean and maintain your ITNs? Do you notice any difference in how easy it is to wash or dry polyethylene versus polyester nets?”*
4. *“Do you think one material (polyethylene or polyester) lasts longer or withstands the elements (e.g., sun, dust, humidity) better than the other?”*
5. *“Are there any maintenance challenges you face with either material (e.g., discoloration, chemical wear)? How does this affect your decision to keep using the nets?”*

**Discussion Point iv:** Perceptions of Efficacy (15-20 minutes**)**

**Objective:** Gain insights into participants' perceptions of the effectiveness of each textile in preventing mosquito bites and protecting against malaria.

1. *“In your experience, how effective do you feel polyethylene and polyester ITNs are in protecting you from mosquito bites?”*
2. *“Do you believe one material is more effective than the other in preventing malaria? Why?”*
3. *“Have you ever noticed a difference in mosquito activity under different types of nets (polyethylene vs. polyester)?”*
4. *“Do you think the material affects the overall strength of the insecticide treatment on the net? How so?”*
5. *“In your community, do people tend to trust one material more than the other when it comes to malaria prevention?”*

**Closing (5-10 minutes)**

1. **Wrap-up and Additional Thoughts:**
   - *“Is there anything else you would like to share about your experiences with insecticide-treated nets (polyethylene or polyester)? Any final thoughts or suggestions for improvement ?”*
2. **Thank You and Next Steps:**
   - Thank the participants for their valuable insights.
   - Inform them of how their feedback will be used to improve ITNs.
   - If applicable, let them know how they can follow up or participate in future research.

**Facilitator Tips :**

- **Active Listening:** Ensure that you actively listen to participants’ answers and probe for more details when needed (e.g., “Can you explain more about why you prefer that material?”).
- **Follow-up Probes:** Use probes like *“Can you tell me more about that?”* or *“Why do you think that is?”* to dive deeper into participants' opinions.
- **Neutral Stance:** Avoid showing personal preferences for any material to prevent bias in responses.
- **Group Dynamics:** Encourage quieter participants to share their thoughts and make sure the discussion remains balanced.
- **Clarification:** Ensure that technical terms (e.g., “polyethylene,” “polyester,” or “ITNs”) are understood by all participants.

**Summary of Key Discussion Points:**

- **Material Preference:** Focus on personal choices between polyethylene and polyester ITNs and why participants prefer one over the other.
- **Comfort and Usability:** Explore how the texture, breathability, and warmth of each material impact comfort, especially in different climates.
- **Durability and Maintenance:** Discuss how each material holds up over time, including ease of cleaning and long-term use.
- **Perceptions of Efficacy:** Understand how each material is perceived in terms of preventing mosquito bites and protecting against malaria.
