# Supplementary material for: Assessing ITN textile preferences: A comparative study of polyethylene and polyester nets across different settings in Burkina Faso
Source: PLoS One. 2025 Aug 22;20(8):e0325580. doi: 10.1371/journal.pone.0325580 (PMC12373252; doi:10.1371/journal.pone.0325580)
Supplement: S2 — (DOCX) [file pone.0325580.s002.docx]

**Additional file 2.** **In-Depth Individual Interview (IDI) Guide and questionnaire: Personal Experiences and Suggestions for ITN Improvements**

**Objective:**
To understand individual experiences with insecticide-treated nets (ITNs), focusing on polyethylene and polyester textiles, as well as cultural, environmental, and practical factors influencing ITN use. Additionally, the interview will explore interviewees' suggestions for improving ITN textiles to better meet their needs.

**Introduction (5-10 minutes)**

1. **Introduction and Purpose :**
   - *“Thank you for taking the time to speak with me today. The purpose of this interview is to learn more about your personal experiences with insecticide-treated nets (ITNs) and the materials they are made from (polyethylene and polyester). We are also interested in understanding any cultural or environmental factors that influence your choice and use of ITNs.”*
   - Explain that the interview is confidential, and their personal experiences and views are valuable to improve ITN usage.
   - Ask for permission to record the interview (if applicable).
2. **Warm-up Questions :**
   - *“Can you tell me a little bit about where you live and your daily routine?”*
   - *“How long have you been using insecticide-treated nets (ITNs), and which type do you currently use?”*

**Main Interview (30-45 minutes)**

**i) Personal Experiences with ITN Textiles (15-20 minutes)**

**Objective:** To explore individual experiences with polyethylene and polyester ITNs, including comfort, texture, and ease of use.

1. **General Experience:**
   - *“Could you describe your general experience with using insecticide-treated nets (ITNs)? How often do you use them?”*
   - *“Which material (polyethylene or polyester) do you currently use, and what made you choose that particular material?”*
2. **Comfort and Texture :**
   - *“How would you describe the comfort of the ITNs you use? Is there any difference in comfort between polyethylene and polyester?”*
   - *“Can you describe the texture of the net? Do you feel one material is softer or rougher than the other?”*
3. **Ease of Use :**
   - *“How easy is it to set up and use the net? Are there any challenges you face when using the net (e.g., installation, sleeping under it)?”*
   - *“Do you feel that the material affects how easy it is to use or maintain the net? For example, does one material feel heavier or harder to handle than the other?”*
4. **Performance in Different Conditions :**
   - *“Have you noticed any differences in how the nets perform in different weather conditions (e.g., hot/dry or humid environments)?”*
   - *“Which material seems to be more breathable or comfortable under your climate conditions?”*

**ii) Cultural and Environmental Influences (15-20 minutes)**

**Objective:** To understand how cultural perceptions and environmental factors in the participant’s region influence their choice and use of ITNs.

1. **Cultural Perceptions :**
   - *“How does your community view the use of insecticide-treated nets (ITNs)? Are there cultural beliefs or practices that influence how people use ITNs?”*
   - *“Do you feel that certain materials are more accepted or preferred in your community (e.g., polyethylene or polyester)? Why do you think that is?”*
2. **Environmental Conditions :**
   - *“How does your local environment (e.g., climate, rural or urban living conditions) affect your choice of ITN material? Are there certain features that are more important due to your environment?”*
   - *“Do you believe that certain materials perform better or worse in your region’s climate? For example, does one material fare better in humidity or heat?”*
3. **Accessibility and Availability:**
   - *“Is it easy to find or obtain insecticide-treated nets (ITNs) in your area? Are there any challenges related to accessing or affording the nets?”*
   - *“Do you think people in your area prefer certain types of nets because of availability or cost?”*

**iii) Suggestions for Improvement (10-15 minutes)**

**Objective**: To gather insights into potential improvements in ITNs, particularly in terms of textile characteristics and adaptations based on personal needs and preferences.

1. **Suggestions for Improvement :**
   - *“If you could make changes to the design or material of insecticide-treated nets (ITNs), what would you change?”*
   - *“Are there any features you think would make the nets more comfortable or easier to use?”*
   - *“What improvements would you suggest for the material itself? For example, would you like it to be softer, more breathable, or more durable?”*
2. **Functionality and Durability:**
   - *“Do you think the nets could be improved in terms of durability? How could they last longer or withstand harsher conditions ?”*
   - *“Would you want the material to be more resistant to damage from the sun, washing, or tearing? How important is that to you?”*
3. **Effectiveness and Protection :**
   - *“How do you think the ITNs could be improved to offer better protection against mosquitoes and malaria?”*
   - *“Do you have any suggestions for improving the insecticide treatment on the nets to make it more effective?”*
4. **Other Adaptations :**
   - *“Are there any other aspects of the nets that could be improved to suit your specific needs (e.g., size, shape, ease of installation)?”*

**Closing (5-10 minutes)**

1. **Wrap-up:**
   - *“Is there anything else you’d like to share about your experience with insecticide-treated nets or suggestions for how they could be improved?”*
2. **Final Thank You :**
   - Thank the participant for their time and valuable insights.
   - Reassure them about the confidentiality of the interview and explain the next steps in the research process.
   - Let the participant know how their feedback will help improve the ITNs.

**Facilitator Tips :**

- **Active Listening:** Listen carefully and ask follow-up questions to dive deeper into the participant’s thoughts (e.g., "Could you tell me more about that?" or "Why do you think that is?").
- **Neutrality:** Avoid showing personal opinions or preferences about any of the ITN materials to ensure unbiased responses.
- **Probing:** Use probes like *“Can you explain more about that?”* or *“How does that make you feel?”* to encourage more detailed responses.
- **Rapport Building:** Foster a comfortable environment where participants feel free to share openly by acknowledging their responses and showing appreciation for their insights.
- **Clarification:** Ensure that technical terms (e.g., “polyethylene,” “polyester,” or “ITNs”) are understood by all participants.

**Summary of Key Themes:**

1. **Personal Experiences with ITN Textiles:** Understand participants’ experiences with both polyethylene and polyester nets, focusing on comfort, texture, and usability.
2. **Cultural and Environmental Influences:** Investigate how cultural perceptions and environmental conditions (e.g., climate, rural vs. urban) impact ITN choice and use.
3. **Suggestions for Improvement:** Gather practical suggestions for enhancing the design, material, and functionality of ITNs, tailored to the specific needs of the community.
